# The Moderating Role of Extroversion and Neuroticism in the Relationship between Autonomy at Work, Burnout, and Job Satisfaction

**DOI:** 10.3390/ijerph17218166

**Published:** 2020-11-05

**Authors:** Jesús Farfán, Marta Peña, Samuel Fernández-Salinero, Gabriela Topa

**Affiliations:** 1 Health Psychology Program, International School of doctorate, National Distance Education University (UNED), 28015 Madrid, Spain; jfarfandiaz@gmail.com (J.F.); martapr87@yahoo.es (M.P.); 2 Psychology Department, Universidad Rey Juan Carlos, 28933 Madrid, Spain; samuel.fernandez@urjc.es; 3 Department of Social and Organizational Psychology, National Distance Education University (UNED), 28040 Madrid, Spain

**Keywords:** burnout, emotional exhaustion, performance, autonomy at work, job satisfaction, neuroticism, extroversion

## Abstract

The main aim of this research project was to determine the relationship that exists between autonomy at work and both burnout and job satisfaction, taking into account the moderating effect of the personality factors extroversion and neuroticism. The study was carried out with 971 volunteers (553 women and 418 men) with a mean age of 37.58 years. The majority had either a university degree (485 participants) or higher education qualifications (Spanish baccalaureate) (202 participants). The following instruments were administered: the Maslach Burnout Inventory (MBI), to measure burnout among participants; the Mini International Personality Item Pool Scale (Mini-IPIP) by Donnellan, Oswald, Baird, and Lucas (2006) to measure the personality factors extroversion and neuroticism; the Brief Index of Affective Job Satisfaction (BIAJS) by Thompson and Phua (2012); and the Job Content Questionnaire (JCQ) by Karasek (1985) to measure autonomy at work. The results obtained indicate that those who enjoy greater autonomy at work have lower levels of emotional exhaustion. The stronger the effect is, the higher the score for extroversion. The personality factors studied were not found to have a direct influence on the criterion variables. However, the interaction effects were significant, except in the case of neuroticism. The results indicate that there are no differences between those who score highly for extroversion and neuroticism and the rest of the population in terms of predicting emotional exhaustion or job satisfaction. The present study aims to serve as a guideline for recruitment specialists, business owners, and job designers, encouraging them to take into account all these variables in order to foster the development of healthy and competitive organizations. Environmental moderators that could interfere with the result have not been introduced in this research. It has focused on the study of the personality factors of the workers, considering that the professional functions performed by the workers were similar.

## 1. Introduction

Ensuring health at work and promoting sustainable organizations are increasingly important challenges in today’s world. Within the field of health promotion, burnout is a variable that has attracted a great deal of attention over recent decades due to the changes that have occurred in work environments [1]. It is essential to understand what causes burnout and to determine the weight of its diverse factors in order to be able to predict it and design interventions focused on those variables that may foster its development. A better understanding of the phenomenon may also help establish satisfactory environments for both managers and employees.

Many studies have associated burnout with stress [2,3], and indeed, burnout can be defined as a chronic occupational stress syndrome [4]. It has also been shown that emotional exhaustion is the key element in burnout [5], making it imperative to find new ways of dealing with this phenomenon. Depersonalization and low levels of personal accomplishment are the other two components of the construct [1].

Some recent studies [6,7,8] have found an inverse relationship between job satisfaction and burnout, although others which have explored this idea in more depth argue that it is in fact certain components of burnout, such as emotional exhaustion, which correlate closely with this variable [9]. Some authors have also found that the relationship between job satisfaction and emotional exhaustion is moderated by contextual variables linked to the work environment [10], thus highlighting the importance of moderator variables, which have been studied very little to date. These limitations highlight the need to develop broader models that reflect the complexity of the work environment.

The present study aims to assess the affective consequences of the work environment, which is why it focuses on emotional exhaustion, which has been shown to be a relevant factor in organizational processes [11].

To fully understand modern-day organizations, it is important to develop interaction models that include both dispositional and situational factors. In this sense, *job demands–resources theory* [12] emphasizes the importance of determining not only the job resources available but also the personal resources upon which the individual can call in order to cope with the demands of their profession. Specifically, job demands refer to any physical, psychological, organizational, or social aspect which requires an effort from the worker [13], whereas job resources are those physical, psychological, and organizational aspects of the job which may (a) reduce the demands of the job and their associated physiological and psychological costs, (b) be decisive in ensuring work-related goals are met, or (c) stimulate personal growth, learning, and development [12]. 

In accordance with this theory, it has been shown that demands are generally linked to processes associated with negative health outcomes or emotional exhaustion [14], whereas resources are linked to processes such as satisfaction and engagement [15], the latter is understood as the employee’s voluntary effort or commitment to the job. Moreover, demands and resources are related to job crafting that focuses on employee job redesign [16].

Recent research [17] suggests that how the individual assesses the environment is a key aspect in determining their level of emotional exhaustion. Long working hours, lack of autonomy, and high levels of interference between work and home life are all factors that may impact employees’ mental health and exacerbate symptoms of emotional exhaustion. The specific nature of each work environment, as assessed by employees, is, therefore, a key factor in determining levels of emotional exhaustion. 

For this reason, some companies are introducing innovations to reduce this problem. The job crafting technique facilitates the adaptation of the worker to the development of their professional tasks [18] because it allows designing the job, adapting it to the way of working of the employee, based on their interests, strengths, and weaknesses. This work system allows reducing emotional exhaustion, increasing performance and productivity.

Karasek’s job demand–control model [19] explains occupational stress in terms of the balance struck between the psychological demands of the job and the level of control perceived by the employee. The model postulates the existence of a significant relationship between occupational stress and health disorders, which are the result of a combination of high psychological demands at work and a low level of control over one’s job. It also posits that a high level of perceived social support reduces the effect of occupational stress, thereby mitigating its adverse consequences. Russell also found a positive correlation between autonomy and job satisfaction [20], and Juárez et al. (2014) concluded that employees’ control over their task was an effective predictor of their occupational health [21]. Autonomy and satisfaction, therefore, seem to play an important role in the development of occupational stress, and jobs can be categorized in accordance with these two factors.

However, the fact that a work environment may be potentially stressful does not necessarily imply that all employees will suffer from burnout. Each person manages stress and interprets the environment in which they work in accordance with the personality factors that characterize them and their own individual life experiences. Jiménez, Hernández, and Gutiérrez (2000) found that stress and burnout arise as a result of the interaction between variables pertaining to the work environment and those pertaining to the individual’s personality. Personality plays an important role in the origin and development of stress and burnout, with those who adopt adequate coping strategies being able to actively engage with their environment and modify it to their advantage [22].

Thus, situational factors may be interpreted differently by different individuals. In this study, individual personality differences are assessed using the big five personality trait model developed by McCrae and Costa (1985) [23]. According to this model, personality is made up of five large dimensions or factors: neuroticism, extroversion, openness to experience, agreeableness, and conscientiousness. In the field upon which we are focused here, several studies have reported a significant relationship between the extroversion and neuroticism factors and emotional exhaustion [24,25] and job satisfaction [9,26], while others have suggested that different relationships exist with job satisfaction, depending on each individual’s specific personality traits [27]. Consequently, we believed it would be interesting to include personality variables in this study. Job satisfaction is one of the most widely studied aspects of the work environment and, to date, significant relationships have been found between it and both occupational performance and occupational health [28], making it a key variable for the development of sustainable organizations.

In light of the above, this study proposes a comprehensive model encompassing both situational and dispositional variables in order to assess their impact on the dependent variables. The main aim of this research project is, therefore, to determine the relationship which exists between autonomy at work and both burnout and job satisfaction, taking into account the moderating effect of the personality factors extroversion and neuroticism. The concepts and variables included in the study are outlined below.

Autonomy at Work. Generally, employees’ control over their jobs has been measured using two different yet closely related theoretical sub-dimensions: creativity and authority to make one’s own decisions, which some authors refer to as autonomy at work [29,30]. The autonomy at work variable is linked to employees’ ability to influence organizational processes and make decisions. 

Moreover, other studies have also highlighted how having greater control over one’s own job leads to greater job satisfaction and reduced stress levels [31]. It is, therefore, interesting to explore how personality traits moderate these relationships in order to come to a deeper understanding of how employees adapt to their jobs.

Emotional Exhaustion. From the beginning, authors studying burnout have posited that an imbalance between job demands and employee resources is a key factor in understanding the impact of the work environment [1]. Since the year 2000, the World Health Organization (WHO) has considered burnout an occupational risk factor and has even highlighted its potential to put workers’ lives in jeopardy. Emotional exhaustion is one of the most important components of burnout and is characterized by loss of energy, fatigue, and the feeling of being worn out. In general, it has been defined as an inadequate means of coping with chronic stress [32]. Peiró (2005) [33] identified an absence of control by the worker as a key factor for understanding burnout, and Jiménez, Hernández, and Gutiérrez (2000) [22] found that health status was closely linked to all the dimensions of burnout (emotional exhaustion, depersonalization, and lack of accomplishment), but in particular to emotional exhaustion. Individuals who scored highly for a resilient personality had lower levels of emotional exhaustion. A resilient personality, therefore, seems to play an important role in reducing the likelihood of suffering from stress and burnout.

Gil-Monte, Peiró, and Valcárcel (1996) [34] found that the dimension which most contributed to frequent feelings of burnout was emotional exhaustion. This finding is consistent with those reported by studies that, using the Maslach Burnout Inventory (MBI), found that emotional exhaustion was the dimension that most impacted burnout [35,36,37]. Similarly, Portero and Vaquero (2015) [9] concluded that emotional exhaustion was a significant variable in the development of burnout among workers. 

Job Satisfaction. Job satisfaction is understood as a positive emotional state that reflects an affective response to one’s job and indicates how individuals feel in relation to the different aspects of their daily work. In short, it is an overall feeling about one’s job and the degree to which one likes it [38,39]. This affective component has been found to be very important in the study of organizations and team management [40]. The factors influencing job satisfaction include income level, work relations, and the employee’s level of control over the decisions that are made [41,42]. 

Occupational stress may affect workers’ mental health, thereby reducing their levels of job satisfaction [43]. Hosseinabadi et al. (2018) [27] found a direct relationship between control over one’s job and job satisfaction, with having the authority to make decisions resulting in employees performing tasks more happily. 

Job satisfaction is also linked to personality variables, such as motivational orientations [43]. We can, therefore, affirm that job demands and control have an impact on affective criterion variables (emotional exhaustion and job satisfaction) in accordance with the subject’s dispositional personality variables, which together determine the perspective from which the work environment is assessed [44].

Extroversion and Neuroticism. Many studies have linked personality traits to the way in which workers carry out their tasks, with the aim of optimizing employee performance [45,46,47,48,49,50,51]. Understanding this relationship is very useful for both recruiting members of staff and assigning them to positions that best fit their personality. It is generally accepted that there are five principal traits or factors that can be used to catalog the structure of each individual personality [52]. Norman (1963) labeled these five main personality factors extroversion, neuroticism, openness to experience, conscientiousness, and agreeableness, and a broad consensus has been reached regarding their validity in the field of personality assessment [53]. 

In the big five personality factor model, extroversion is the dimension that measures sociability. It is linked to positive strategies for coping with aversion, as well as to sociability, assertiveness, and activity [54]. In the world of work, it has been suggested that extroversion is a variable that predicts adjustment in jobs requiring interaction and cooperation. Several studies have explored the relationship between extroversion and burnout among employees, with Swider and Zimmerman (2010), for example, finding that those who scored lower for extroversion were more likely to experience this syndrome than those who scored highly [55]. Emotional stability is strengthened in extroverts, or those who enjoy interacting with other people, thereby boosting their resistance to burnout [56]. For their part, Meymandpour and Bagheri (2017) found that when employees worked mainly from home (teleworking), those scoring lower for extroversion were more likely to experience burnout [24]. 

In the big five personality factor model, neuroticism is the dimension that measures emotional instability. It is linked to anxiety, depression, irritability, worry, and insecurity and seems to be an effective predictor of performance in a wide variety of different jobs [54]. Some authors have found a close positive association between neuroticism and burnout [25], as well as an inverse relationship between neuroticism and job satisfaction [57].

**Figure 1:**
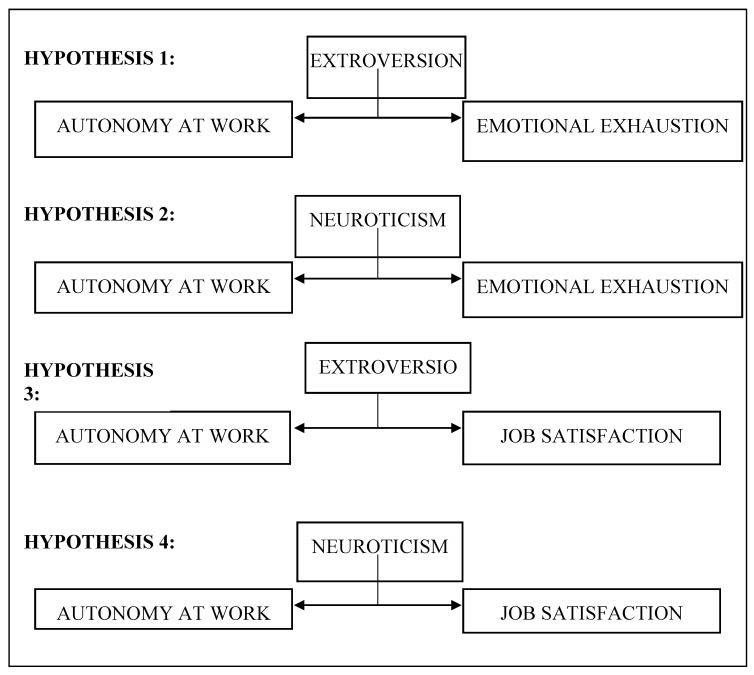
Study hypotheses.

It is interesting to explore the moderating role of extroversion and neuroticism in the relationship between autonomy at work, burnout, and job satisfaction since this may provide information about workers’ future job performance. Thus, the main aim of this research project is to assess the relationship between autonomy at work and both burnout and job satisfaction, taking into account the moderating effect of extroversion and neuroticism (Figure 1). 

The initial hypothesis on which the study is based is that interaction models that encompass both situational and dispositional variables will help researchers gain a fuller understanding of organizational dynamics. The working hypotheses proposed are as follows:

**Hypothesis** **1** **(H1):** *The negative relationship between autonomy at work and emotional exhaustion is moderated by extroversion.*

**Hypothesis** **2** **(H2):** *The negative relationship between autonomy at work and emotional exhaustion is moderated by neuroticism.*

**Hypothesis** **3** **(H3):** *The positive relationship between autonomy at work and job satisfaction is moderated by extroversion.*

**Hypothesis** **4** **(H4):** *The positive relationship between autonomy at work and job satisfaction is moderated by neuroticism.*

## 2. Method

### 2.1. Sample

The sample comprised 971 people (553 women and 418 men) with a mean age of 37.58 years. The sample was obtained through a no probabilistic convenience sampling procedure. The majority had either a university degree (485 participants) or higher education qualifications (Spanish baccalaureate) (202 participants) and all participated in the study voluntarily. Related to the job type, we collected data about the employment category, and our sample was composed of 95 individuals (9.8%) occupying manager positions. Besides, 230 individuals (23.7%) were occupying middle-level management positions, and 482 individuals (49.6%) were occupying technical and administrative positions. Moreover, our sample was composed of 164 individuals (16.9%) who were occupying non-qualified organizational positions. The questionnaire was administered individually after each participant had given their informed consent. The data obtained were analyzed using the SPSS statistical package (IBM, Armonk, NY, USA) for Windows 21.0.

### 2.2. Instruments

The *Mini International Personality Item Pool Scale (Mini-IPIP)* [58] was used to assess extroversion and neuroticism. This instrument comprises 100 items that together define five personality domains: Emotional Stability or Neuroticism (ES), Extroversion (E), Intellect (I), Agreeableness (A), and Conscientiousness (C). Each domain is measured through 20 items, drafted as statements describing typical behaviors. Respondents are asked to rate the accuracy of each statement as it pertains to them on a five-point response scale ranging from *very inaccurate* to *very accurate* [59]. The Job Content Questionnaire (JCQ) [60] was used to measure autonomy at work. The JCQ is an objective instrument that assesses psychosocial risks. It comprises 29 items with widely demonstrated reliability and validity [61] which assess psychological demands, decision latitude, and social support (from colleagues and supervisors).

The Maslach Burnout Inventory (MBI) [62] is an instrument that operationalizes burnout, establishing the following dimensions as assessment criteria: emotional exhaustion, depersonalization, and low personal accomplishment. In this study, the Spanish version of the instrument [63] was used to assess the emotional exhaustion dimension. 

Job satisfaction was assessed using the Brief Index of Affective Job Satisfaction (BIAJS) [42]. This instrument has been validated in terms of its internal consistency, temporal stability, criterion and convergent validity, and invariance between both populations with different nationalities and those that perform different kinds of jobs. 

All the instruments were validated previously and have demonstrated their suitability on previous organizational field researches [64,65]. 

### 2.3. Results

First, we conducted an exploratory analysis of all the variables included in our research. Based on the Kolmogorov–Smirnov test, we confirmed that our data were normally distributed and our statistical analysis was appropriated. Kolmogorov–Smirnov values above 0.05 indicate normal distribution. In the descriptive analysis of the 971 participants, a mean of 3.64 (SD 0.90) was obtained in relation to the predictor variable (autonomy at work). The mean obtained for the first criterion variable (emotional exhaustion) was 2.56 (SD 0.85) and for the second criterion variable (job satisfaction) it was 3.45 (SD 0.99). As for the moderator variables, the means obtained were 2.92 (SD 0.66) for extroversion and 2.77 (SD 0.77) for neuroticism.

The correlational analysis (Table 1) revealed that autonomy at work correlated significantly with both criterion variables, with this association being negative in the case of emotional exhaustion and positive in the case of job satisfaction. Moreover, the two criterion variables correlated significantly, and negatively, with each other.

No significant correlation was observed between extroversion and neuroticism. Extroversion correlated significantly, and positively, with autonomy at work and job satisfaction, and negatively with emotional exhaustion. For its part, neuroticism correlated significantly, and negatively, with job satisfaction and autonomy at work and significantly, and positively, with emotional exhaustion.

In the regression model, when emotional exhaustion was used as the criterion variable, all the variables were found to be significant: autonomy at work (t = −16.22 *p* = 0.000), extroversion (t = −7.37 *p* = 0.000), and neuroticism (t = 5.31 *p* = 0.000). The same result was observed also when job satisfaction was used as the criterion variable: autonomy at work (t = 17.78 *p* = 0.000), extroversion (t = 4.56 *p* = 0.000), and neuroticism (t = −5.13 *p* = 0.000).

As regards the analysis carried out with Hayes’ PROCESS macro [66], the simple moderation model (model 1) was used with the quantitative moderator variable. In all cases, three coefficients were obtained: b2, which estimated the main effect of the moderator variable (M) on the dependent variable (DV or Y); b1, which estimated the effect of the independent variable (IV or X) on the DV; and b3, which estimated the interaction effect on the DV.

In the first two hypotheses (H1 and H2), X represented autonomy at work and Y emotional exhaustion. In relation to the first hypothesis (H1), which posited that “the relationship between autonomy at work and emotional exhaustion is moderated by extroversion, the PROCESS macro established three scores for the moderator variable (extroversion): low (2.26), medium (2.92), and high (3.58). The three coefficients obtained in the regression analysis for H1 revealed that the effect of extroversion was not significant (B (extroversion) = 0.01, *p* < 0.9574), the effect of the IV was not marginally significant (B (autonomy at work) = −0.22, *p* < 0.0334), and the interaction was significant (B (autonomy at work x extroversion) = −0.07, *p* < 0.0360).

When the conditional effect of the IV on the DV was analyzed, the results revealed that the effect of autonomy at work on emotional exhaustion was statistically significant in all three extroversion scores, in other words, among those with low (x → Y/M = 2.26 = −0.39, *p* < 0.000), medium (x → Y/M = 2.92 = 0.44, *p* < 0.000), and high levels of extroversion (x → Y/M = 3.58 = −0.49, *p* < 0.000). 

For the second hypothesis: “the negative relationship between autonomy at work and emotional exhaustion is moderated by neuroticism”, three scores were established for the moderator variable (neuroticism): low (2.00), medium (2.78), and high (3.55).

The three coefficients obtained in the regression analysis for H2 revealed that the effect of neuroticism was not significant (B (neuroticism) = 0.13, *p* < 0.2914), the effect of the IV was not marginally significant (B (autonomy at work) = −0.47, *p* < 0.0000), and the interaction was not significant (B (autonomy at work x neuroticism) = 0.01, *p* < 0.7315). We can, therefore, present no evidence which attests to the validity of this hypothesis.

When the conditional effect of the IV on the DV was analyzed, the results revealed that the effect of autonomy at work on emotional exhaustion was statistically significant in all three neuroticism scores, or in other words, among those with low (x → Y/M = 2.00 = −0.44, *p* < 0.000), medium (x → Y/M = 2.78 = −0.44, *p* < 0.000), and high levels of neuroticism (x → Y/M = 3.55 = −0.43, *p* < 0.000). 

In the last two hypotheses (H3 and H4), X represented autonomy at work and Y job satisfaction. Hypothesis 3 (H3) posited that: “the positive relationship between autonomy at work and job satisfaction is moderated by extroversion” and the PROCESS macro established three scores for the moderator variable (extroversion): low (2.26), medium (2.92), and high (3.58). 

The three coefficients obtained in the regression analysis for H3 revealed that the effect of extroversion was not significant (B (extroversion) = −0.13, *p* < 0.3838), the effect of the IV was not marginally significant (B (autonomy at work) = 0.30, *p* < 0.0151), and the interaction was significant (B (autonomy at work x extroversion) = 0.09, *p* < 0.0291).

When the conditional effect of the IV on the DV was analyzed, the results revealed that the effect of autonomy at work on job satisfaction was statistically significant in all three extroversion scores, in other words, among those with low (x → Y/M = 2.26 = 0.50, *p* < 0.000), medium (x → Y/M = 2.92 = 0.56, *p* < 0.000), and high levels of extroversion (x → Y/M = 3.58 = 0.62, *p* < 0.000). 

Hypothesis 4 (H4) posited that: “the positive relationship between autonomy at work and job satisfaction is moderated by neuroticism”. Three scores were established for the moderator variable (neuroticism): low (2.00), medium (2.78), and high (3.55).

The three coefficients obtained in the regression analysis for H4 reveal that the effect of neuroticism was not significant (B (neuroticism) = 0.05, *p* < 0.6836), the effect of the IV was not marginally significant (B (autonomy at work) = 0.73, *p* < 0.0000), and the interaction was not significant (B (autonomy at work x neuroticism) = −0.07, *p* < 0.0694).

When the conditional effect of the IV on the DV was analyzed, the results revealed that the effect of autonomy at work on job satisfaction was statistically significant in all three neuroticism scores, or in other words, among those with low (x → Y/M = 2.00 = 0.60, *p* < 0.000), medium (x → Y/M = 2.78 = 0.55, *p* < 0.000), and high levels of neuroticism (x → Y/M = 3.55 = 0.50, *p* < 0.000).

## 3. Discussion

The main aim of this study was to examine the moderating role of extroversion and neuroticism in the relationship between autonomy at work, burnout, and job satisfaction.

The results obtained indicate that the personality variables studied had no direct influence on the criterion variables. However, the interaction effects were significant, except in the case of neuroticism. On the basis of these findings, we can, therefore, conclude that there are no differences between those who score highly for extroversion and neuroticism and the rest of the population in terms of predicting emotional exhaustion or job satisfaction. 

However, the results also suggest that those who enjoy a greater degree of autonomy in their job have lower levels of emotional exhaustion, which in turn may foster their motivation and performance at work. Moreover, the effect size of this association was observed to increase the higher score for extroversion, perhaps due to the fact that more extroverted individuals express their emotional states more explicitly, something which helps them adapt better to their environment. Some authors [67] have linked certain pathologies to emotional internalization, which may, in turn, affect job performance. 

The results of the present study reveal that the moderator effect of neuroticism was not significant in either the direct relationship between variables or their interaction. Variations in neuroticism scores had very little impact on B coefficients, indicating that neuroticism may be related to other methods of coping with the contextual variables of the work environment. Given that previous studies have found different findings [68], we would suggest that one possible explanation may be that those who score highly for neuroticism tend to rationalize things more, and as previous research has shown, this personality trait is linked also to different failures and dynamics in emotion regulation [69].

For its part, many studies have found job satisfaction to be associated with other physical and mental health variables, self-esteem, and burnout [70,71], indicating that it is a very important variable to promote. As with the other criterion variable (emotional exhaustion), in the present study, personality variables were not found to have any significant direct relationship with job satisfaction, although a statistically significant direct influence of autonomy at work on job satisfaction was observed. As regards the moderating effect of extroversion, higher scores for this personality trait were found to be associated with higher B levels. Extroverts are characterized by their tendency to express their emotional states more explicitly.

Finally, neuroticism was not found to have a significant moderating effect on the relationship between autonomy at work and job satisfaction. In other words, neuroticism does not moderate the relationship between these two variables. It may be that neuroticism is more involved in the moderation of cognitive factors, although further research is required to clarify this. 

The present study provides greater insight into the relationships established between autonomy at work and emotional variables such as emotional exhaustion and job satisfaction. The results obtained also increase our understanding of which personality variables influence and moderate the direct relationships observed, and the direction of this moderation. For example, extroversion was observed to act as a moderator, strengthening the effects of the direct relationships observed in the sample.

The study also has certain limitations. Firstly, it follows a correlational cross-sectional design. It may be interesting to conduct a longitudinal study to further explore and explain the relationships which exist between the variables studied over time. Secondly, the sample used was limited since it contained a large percentage of people with a high education level. Future research may wish to recruit samples with a greater range of academic qualifications in order to determine whether or not the results vary accordingly. Thirdly, the sample was not selected through a probabilistic procedure. Since our sample is not representative, it is recommended to replicate the research using randomized sampling procedures. Moreover, we recommend collecting data about different jobs or sectors in order to assess if there are changes in the variables posed in this research and in their relationships.

On the other hand, research focused on personality factors and environmental factors characteristics of the job has not been taken into account, which can provide incomplete results. It would be convenient for future research to take these variables into account.

## 4. Conclusions

In today’s world, companies are constantly searching for ways to increase their efficacy and efficiency in order to ensure the best possible results. One important aspect that should be taken into consideration in this search is the fact that jobs should adapt to the daily working dynamics and qualities of each employee, in accordance with their abilities and personality. This will, in turn, enhance their wellbeing, motivation, and commitment, thus helping the company improve its overall performance.

Organizations are becoming increasingly aware of the benefits of ensuring the wellbeing of all their employees and are gradually adapting their structures to enable workers to perform their tasks with a greater degree of freedom, creativity, and autonomy, setting their own pace and customizing their specific jobs. This process, in which employees influence the nature and characteristics of their job, is known as job crafting. As Robledo, Zappalá, and Topa point out, this concept has been developed in order to enable a better understanding of employees’ cycle of wellbeing and the positive results obtained by their organization [72]. 

The present study aims to serve as a guideline for recruitment specialists, business owners, and job designers, encouraging them to take into account how personality factors, autonomy at work, and job satisfaction are related to employee performance and burnout in order to foster the development of healthy and competitive organizations.

## Figures and Tables

**Table 1:** Pearson correlation matrix between the study variables.

	1	2	3	4	5
1. Extraversion	1				
2. Neuroticism	−0.037	1			
3. Autonomy at work	0.075 *	−0.128 **	1		
4. Emotional exhaustion	−0.239 **	0.209 **	−0.478 **	1	
5. Job satisfaction	0.165 **	−0.206 **	0.512 **	−0.842 **	1

* *p* < 0.05; ** *p* < 0.01.
